# Interferon-alpha-based immunotherapies in the treatment of B cell-derived hematologic neoplasms in today’s treat-to-target era

**DOI:** 10.1186/s40164-017-0081-6

**Published:** 2017-07-14

**Authors:** Li Zhang, Yu-Tzu Tai, Matthew Zhi Guang Ho, Lugui Qiu, Kenneth C. Anderson

**Affiliations:** 1 LeBow Institute for Myeloma Therapeutics and Jerome Lipper Center for Multiple Myeloma Center, Dana-Farber Cancer Institute, Harvard Medical School, Boston, MA USA; 2 Department of Hematology, West China Hospital, Sichuan University, Chengdu, Sichuan China; 3 UCD School of Medicine, College of Health and Agricultural Science, Belfield, Dublin Ireland; 4 UCD Conway Institute of Biomolecular and Biomedical Research, University College Dublin, UCD, Belfield, Dublin Ireland; 5 State Key Laboratory of Experimental Hematology, Institute of Hematology & Blood Diseases Hospital, Chinese Academy of Medical Science & Peking Union Medical College, Tianjin, China

**Keywords:** Interferon-alpha, CD38, CD20, Lymphoma, Multiple myeloma, Targeted therapy, Bone marrow microenvironment

## Abstract

B cell lymphoma and multiple myeloma (MM) are the most common hematological malignancies which benefit from therapeutic monoclonal antibodies (mAbs)-based immunotherapies. Despite significant improvement on patient outcome following the use of novel therapies for the past decades, curative treatment is unavailable for the majority of patients. For example, the 5-year survival of MM is currently less than 50%. In the 1980s, interferon-α was used as monotherapy in newly diagnosed or previously treated MM with an overall response rate of 15–20%. Noticeably, a small subset of patients who responded to long-term interferon-α further achieved sustained complete remission. Since 1990, interferon-α-containing regimens have been used as a central maintenance strategy for patients with MM. However, the systemic administration of interferon-α was ultimately limited by its pronounced toxicity. To address this, the selective mAb-mediated delivery of interferon-α has been developed to enhance specific killing of MM and B-cell malignant cells. As such, targeted interferon-α therapy may improve therapeutic window and sustain responses, while further overcoming suppressive microenvironment. This review aims to reinforce the role of interferon-α by consolidating our current understanding of targeting interferon-α with tumor-specific mAbs for B cell lymphoma and myeloma.

**Electronic supplementary material:**

The online version of this article (doi:10.1186/s40164-017-0081-6) contains supplementary material, which is available to authorized users.

## Background

B-cell neoplasms account for about 80% of lymphomas, which are the most common type of blood cancer. In late 1990s, a new era of monoclonal antibody (mAb)-based immunotherapy emerged with the first anti-CD20 mAb for treatment of B-cell lymphomas. Surface CD20, a pan B-cell marker, is expressed during most stages of B cell development: on late pro-B cells to naïve, mature, and memory B cells; but not on precursor B cells, early pro-B cells, plasma blasts or plasma cells. Accordingly, anti-CD20 mAbs directly deplete B cells of intermediate stages whilst sparing pre-B cells and long-lived plasma cells, which highly expressed cell-surface CD38 instead. Multiple myeloma (MM), the second most common blood cancer, is a distinct B-cell derived neoplasm characterized by expansion of plasma cells in bone marrow. The mAb therapies have become available for MM patients by targeting SLAM Family Member 7 (SLAMF7) [1, 2] and CD38 [3], both of which highly express on primary MM cells. Specifically, anti-CD38 mAb daratumumab is the first mAb showing activity as a monotherapy in MM [3]. A very recent interim analysis of the phase 3 CASTOR trial also showed that therapeutic anti-CD38 mAb, when combined with bortezomib and dexamethasone, can significantly prolong progressive-free survival (PFS) in patients with early relapsed and/or refractory MM [4]. Although these mAb-based immunotherapies have led to significant improvements in treatment of B-cell neoplasms, patients may relapse and ultimately succumb to the disease. Therefore, the timely identification of an alternative approach, which more effectively destroys tumor cells including cancer stem cells (CSCs), is urgently needed.

Significant research efforts spanning nearly 4 decades have explored usage of cytokines as immunomodulators to enhance host immune response against cancer. The most commonly used cytokines are the interferons (IFN), named in 1957 based on their ability to “interfere” with viral replication in infected cells [5]. IFNs were then further classified into 3 pleiotropic polypeptides: type I, II, and III (Table 1) [5, 6]. In 1978, type I IFNs from supernatant of human leukocytes exposed to viruses were purified to homogeneity and sequenced, leading to the discovery of various subtypes of IFNs [7]. In 1981, recombinant IFNs were successfully expressed by Genentech, allowing for the large scale production of “clean” IFNs to meet both research and clinical demands [8, 9]. Interferon-alpha (IFN-α), a type I IFN, is the first recombinant subtype and also the most commonly used IFN in anti-cancer therapy. IFN-α comprises a family of more than 20 related but distinct members encoded by a cluster on chromosome 9. Among these, the most frequently used is IFN-α2, having 3 recombinant variants (α2a, α2b, α2c) depending upon the cells of origin [10]. IFN-α2b is the predominant variant in human genome.

**Table 1:** Subtypes of IFN family

Type	Class (no. of subtypes)	Chromosomal loci	Receptor	Receptor’s chromosomal	Commercially-available recombinant products (trade name)	Cellular source	Inducing agent	Major activity	Clinical application
I^a^	α (16)	9p	IFNAR1 IFNAR2	21 21	IFNα-2a (Roferon A) IFNα-2b (Intron A, Reliferon, Uniferon) PEGylated IFNα-2a (Pegasys, Reiferon Retard) PEGylated IFNα-2b (PegIntron, Pegetron)	Leukocytes (especially pDCs^b^), lymphoblastoid cells	Viruses; dsRNA, B-cell mitogens, foreign cells, tumor cells	Anti-tumor, anti-viral	Anti-tumor (hematological malignancies such as leukemia and lymphomas, solid tumors such as melanoma and Kaposi sarcoma), anti-vira (hepatitis B and C)
	β (2)				IFNβ-1a (Rebif, Avonex, Cinnovex) IFNβ-1b (Betaseron/Betaferon)	Fibroblasts, epithelial cells	Viruses; dsRNA	Balances pro- and antiinflammatory agents in The brain	FDA approved for treatment of multiple sclerosis (MS)
II	γ (1)	12q12	IFNGR1 IFNGR2	6 21	IFNγ-1b (Actimmune)	CD4 and CD8 T cells, NK cells, NKT cells, macrophages, DCs, B cells	Mitogenic or antigenic agents	Immunoregulation; potent phagocyte-activating effects and enhancement of ADCC And NK activity	FDA approved for treatment of chronic granulomatous disease (TB, mycosis) and osteopetrosis
III	λ (3)	19	IFNLR1 IL10R2	1 21	PEGylated IFNλ-1a	pDCs	IFNα, IFNλ, viruses	Anti-tumor anti-viral	Phase II clinical trial as anti-viral agent in chronic HBV infection

*IFN* interferon, *FDA* US Food and Drug Administration, *ADCC* antibody-dependent cell-mediated cytotoxicity, *NK* natural killer, *NKT* natural killer T, *pDCs* plasmacytoid dendritic cells

^a^Type I interferons also include IFN-*κ*, IFN-δ, IFN-ε, IFN-τ, IFN-ω and IFN-ζ,which are currently not being used clinically in humans and thereby excluded in the table

^b^Most cells can secrete IFN-α, but pDCs have the greatest capacity

IFN-α can be secreted by intratumoural dendritic cells (DCs) and malignant cells in response to various stimuli and via positive autocrine and paracrine loops. As reported, a majority of human B-lineage cell lines (e.g. lymphoblastoid cells, B lymphoma, and MM cells) spontaneously produce significant amounts of IFN-α [11]. Plasmacytoid DCs have earned the moniker “human IFN-producing cells” (IPCs), hence they have the greatest capacity to secrete type I IFNs. In the classical model of an antiviral immune response, IPCs are involved at two stages: (1) during the initial innate immune response stage, IPCs rapidly secrete type I IFNs to promote the function of natural killer (NK) cells, B cells, T cells, and myeloid DCs, and (2) at a later stage involving the adaptive immune response, IPCs differentiate into mature DC, which in turn directly regulates the function of T cells. All known IFN-α subtypes exert their function through a specific cell surface membrane receptor complex known as IFN-α receptor (IFN-ΑR), commonly designated as IFN α/β receptor. IFN-ΑRs consist of two high affinity chains: a 110 kDa subunit α (IFN-ΑR1) reported in 1990; and a 102 kDa subunit β (IFN-ΑR2c) reported in 1994. Additionally, two different spliced isoforms of IFN-ΑR subunit β have been reported: (1) 40 kDa soluble IFN-ΑR2a; and (2) 5 kDa transmembrane short form, IFN-ΑR2b. IFN-α binding to IFN-ΑRs leads to activation of intracellular signaling cascades that increase the expression and promote the activation of signal transducers and activators of transcription (STAT)1, STAT2, and STAT3. STAT1 is required for IFN-α-mediated cell death. IFN-ΑRs are expressed not only on malignant cells but also on non-malignant cells, which contributes to anti-tumor effects and nonspecific toxicity by IFN-α.

Systemic IFN-α administration is, to a large extent, hampered by its short half-life, high myelotoxicity, and paradoxical immunosuppressive effects. At present, cell-based immunotherapy is a very promising therapeutic approach; incorporation of a cell-based approach that exploits the specificity of mAb-targeting can selectively deliver IFN-α into the tumor compartment, with fewer side effects as normal cells are spared. This review thereby reevaluates the utility of IFN-α-based regimens for B-cell lymphoma and MM in the current treat-to-target era.

## Preclinical studies of IFN-α in B cell lymphoma and myeloma

Recombinant IFN-α has shown activity against B-cell hematologic neoplasms, primarily through indirect depletion of B-cell neoplasms by immune activation of IFN-ΑR-expressing immune effector cells [12]. For T cells, IFN-α induces the generation and long-term survival of both cytotoxic CD8+ T cell (CTL) and memory CD8+ T cells against tumor antigens, as well as polarizes immune responses towards CD4+ T helper-1 (Th1) phenotype. For NK cells, IFN-α enhances NK cell-mediated toxicity and survival of NK cells. For B cells, IFN-α positively regulates antibody production. For DC cells, IFN-α promotes their maturation and chemotaxis. Moreover, IFN-α treatment induces the expression of programmed cell death-1 (PD-1) on tumor-infiltrating T cells and PD-L1 on tumors [13], which can be neutralized using checkpoint blockade with anti-PD-1/PD-L1 mAbs [14, 15], currently in clinical trials in both lymphoma and MM.

IFN-α can also trigger direct anti-tumor cytotoxicity. By activating IFN-ΑR signaling in B cell lymphomas, IFN-α can induce apoptosis [16], inhibit proliferation [17] and cell cycle progression [18], and promote terminal differentiation in cancer cells [19]. IFN-α signaling also upregulates major histocompatibility complex class I molecules on the surface of tumor cells, leading to enhanced tumor recognition by CTLs. IFN-α was recently reported to upregulate the expression of tumor-associated antigens on human breast cancer xenografts, highlighting their potential for synergy with mAb therapy [20].

However, the precise mechanisms underlying IFN-α’s anti-myeloma effect remain unclear [21]. This is due, in part, to contradictory reports on the effects of IFN-α on ex vivo cultured myeloma cells: some studies showed that IFN-α induces apoptosis and inhibits growth on myeloma cell lines [22], while other studies reported that IFN-α is a survival factor for human myeloma cells via upregulation of anti-apoptotic molecule Mcl-1 [23]. There is an ongoing debate questioning relevance of an ex vivo system to model the highly complex tumor microenvironment in MM [24]. The utility of IFN-α as a maintenance drug for patients with MM was first reported in 1990 [25]. Since then, multiple studies to define the therapeutic benefit of IFN-α-based maintenance regimens have had conflicting results. The primary focus of maintenance therapy in MM is to improve PFS and overall survival (OS). The achievement of positive responses hinges on the thorough depletion of CSC pools (i.e. myeloma-initiating cells) after minimal residual disease (MRD) is achieved following induction therapy. Residual myeloma cells can survive by senescence and entering into the quiescent G0 phase of the cell cycle [26], while IFN-α initially induced cell cycle arrest at the G0/G1 phase in an in vivo mouse model. Thereby, chronic stimulation by IFN-α could cause dormant hematopoietic stem cells to efficiently exit G0, and reverse therapeutic-induced senescence and drug-resistance [27]. IFN-α can therefore exert direct anti-cancer effects by re-activating and mobilizing senescent CSCs [28], which may explain the efficacy of IFN-α maintenance therapy in MM.

## History of IFN-α-based therapy in B cell lymphoma and myeloma

In the field of B cell lymphoma and MM, the usage of IFN-α-based immunotherapies spans two distinct eras: (1) pre-mAb; and (2) post-mAb eras (Table 2). The utilization of IFN-α in the treatment of human B cell lymphoma dates back to the late 1970s, beginning with the use of natural IFN-α in murine models of leukemia and lymphoma (Additional file 1: Table S1) [29–31]. In 1981, the National Cancer Institute undertook Phase II trials of IFN-α2a in patients with low-grade non-Hodgkin’s lymphoma (NHL) [32]. And then, IFN-α has been used mainly in the treatment of low-grade follicular lymphoma (FL), the most common indolent NHL. The efficacy of IFN-α in cutaneous T cell lymphoma (CTCL) was first reported in 1984 and also subsequently at the 1995 International Conference on CTCL to be the most effective single agent treatment. However, initial results of IFN-α treatment of other B-cell neoplasms were far less impressive, since IFN could provide only palliative benefit in certain low-grade or early stage B-cell lymphomas, with complete remission and overall response of 10 and 48%, respectively [33, 35]. The first reported instance of IFN-α use in human MM dates back to 1979 when Mellstedt et al. demonstrated its efficacy in previously untreated myeloma [36]. Since then, IFN-α2b has achieved 50 and 15% responses in patients with newly diagnosed and refractory MM, respectively [37]. From 1997 onwards, the introduction of anti-CD20 mAbs led to significantly better disease control in high-grade lymphoma subtypes (e.g. diffuse large B cell lymphoma) and advanced stage/high-grade FL [38]. Increased survival, due to the use of rituximab, has changed disease course to a more indolent one, affording time to define the effect of IFN-α treatment of aggressive lymphomas as part of an induction and maintenance strategy [39]. However, these studies are mostly single arm, due to the difficulty in obtaining a large sample size related to high mortality in these high-risk populations.

**Table 2:** Milestones of IFNs use for B cell malignancies

Year	Disease	Milestone	Type of IFN	References
		IFNs as anti-viral agent		
1957	Virus	Discovery of IFNs as a broad spectrum antiviral protein	Crude^a^	Isaacs and Lindenmann [5]
		IFNs as anti-cancer agent in the pre-immunotherapy era		
1963	Leukemia	First reported use of IFNs in human cancer: AML	Crude	Falcoff et al. [29]
1974	Osteosarcoma	IFNs in first large scale clinical trial (nonrandomised; 83 patients; Sweden) in human cancer	Crude	Strander et al. [30]
1977	Lymphoma	First reported use of IFNs in treatment of Lymphoma (diffuse histiocytic lymphoma)	Crude	Merigan et al. [31]
1978	MM	First reported use of IFNs in MM	Crude	Mellstedt et al. [36]
1978–1980	–	1978: IFN was purified to homogeneity and two subtypes of IFN (alpha and beta) were then sucessful purified	Purified	
		1980: Nomenclature formally adopted classifying IFNs into 3 categories based on antigenic specificity (alpha, beta, gamma)		
1981	–	First successful expression of immune IFNs by recombinant DNA	Recombinant	Genentech [8]
1981	Lymphoma	National cancer institute carried out Phase II trials of IFN-α-2a in NHL cases	Recombinant	Foon et al. [32]
The middle	Hairy cell	A major breakthrough that the effectiveness of IFNs was found in hairy cell leukemia		NEJM, 1984,15;791; AM J MED, 1986,351; BLOOD, 1985,1017; BLOOD, 1985,644; JCO, 1986,900
1980s	leukemia			
1980s–1990s	Lymphoma	Conflicting results of clinic trials on the efficacy of IFNs in survival of indolent lymphoma: (1) IFN monotherapy, (2) IFN combined cytoreductive chemotherapy, (3) IFN-contained maintenance	Purified/recombinant	Oncology [33]
2000	MM	Meta-analysis of 30 randomised trials looking at use of IFNs in MM: (1) For induction therapy (2333 patients; 17 trials), IFNs resulted in 6.6% higher response rates, and prolonged relapse free and overall survival (2) For maintenance therapy (1615 patients; 13 trials), IFNs led to prolongation of relapse-free and overall survival	Recombinant	Fritz and Ludwig [37]
		IFNs as anti-cancer agent in the post-immunotherapy era		
Early-mid				
2000s	Lymphoma	Rituximab with CHVP + IFNs for follicular lymphoma patients by GELA-GOELAMS FL200	Recombinant	Salles et al. [38]
2008	Lymphoma	IFNα-2a+ rituximab maintenance in a Phase II study	Recombinant	Nordic Lymphoma Group [39]
2010	MM	Interferon-antibody fusion proteins (refer to Table 3)	Recombinant	

*IFN* interferon, *AML* acute myeloid leukemia, *MM* multiple myeloma, *DNA* deoxyribonucleic acid, *NHL* non-Hodgkin lymphoma

^a^ *Crude IFNs* prepared by harvesting interferon secreted by primary cells infected with viruses, resulting in preparations that were less than 1% IFNs by weight (highly impure)

## IFN-α-targeted immunocytokines in B cell lymphoma and myeloma

Although higher doses of IFN-α demonstrate greater anti-tumor activity, its significant systemic toxicities result in a very narrow therapeutic index (low maximum tolerated dose vs high optimal therapeutic dose). To address this limitation, several strategies have been explored to selectively deliver IFN-α to the tumor itself, including: (1) immunocytokines; (2) genetically modified DCs expressing IFN-α; (3) viral and other tumor-targeting vectors encoding IFN-α [40–43]; and (4) vectors encoding pattern recognition receptor agonists delivered directly into tumor microenvironment. One major strategy currently under pre-clinical development aims to target IFN-α to specific cell populations (such as malignant cells or specific types of leukocytes) by conjugating IFN-α to mAbs to generate antibody-based IFN-α fusion proteins, also called immunocytokines or immunoconjugates.

**Fig. 1:**
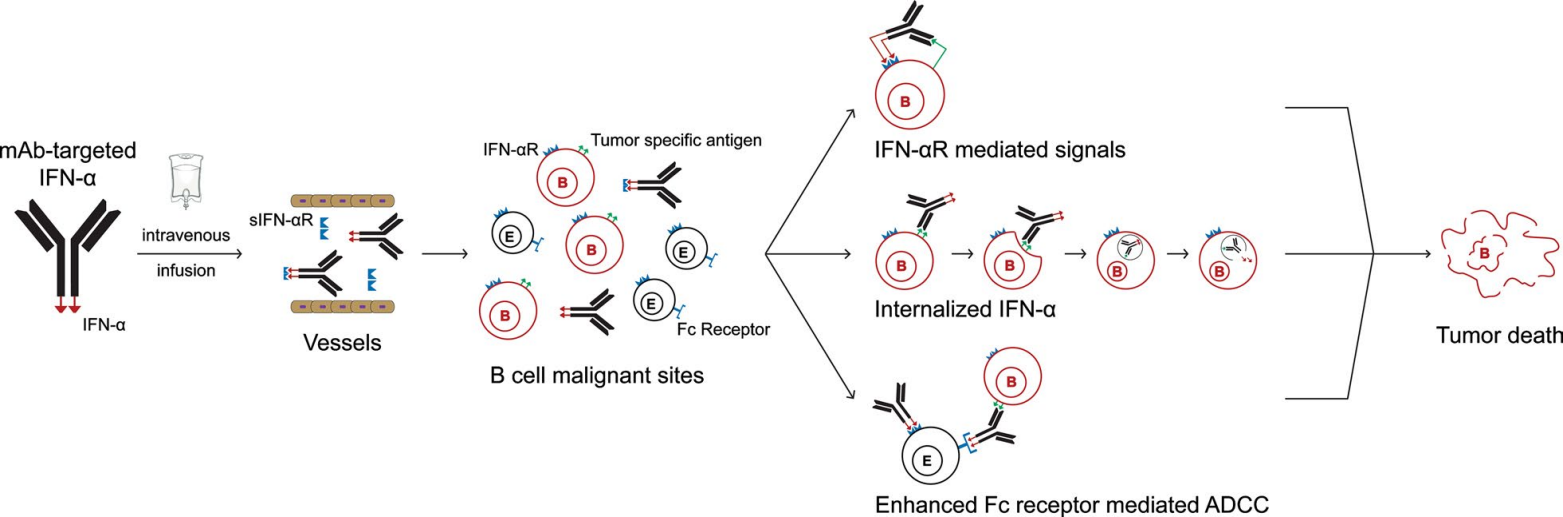
Enhancement of anti-tumor immunity by antibody-targeted IFN-α in B cell malignancies. Antibody-IFN-α fusion proteins are given by intravenous administration. The delivery of concentrated quantities of IFN-α to malignant sites is facilitated by tumor specific mAbs. Three potentially important mechanisms used by antibody-IFN-α fusion proteins to kill targeted tumor cells are: (1) IFN-αR mediated signals, i.e., IFN-α binds to membrane receptor IFN-αR expressed on tumor cells and activates downstream pathways to induce apoptosis; (2) IFN-α internalization, i.e., after mAb-IFN-α fusion proteins are internalized, IFN-α is released within cancer cells; (3) enhancing Fc receptor mediated ADCC, i.e., IFN-α augments ADCC exerted by mAbs through binding to the membrane receptor IFN-αR expressed on effector cells. *E* effector cells including NK cells, γδ T cells, macrophages and dendritic cells, *B* malignant B cells, *IFN* interferon, *sIFN-αR* soluble interferon alpha receptor, *mAb* monoclonal antibody, *ADCC* antibody-dependent cell-mediated cytotoxicity

The potential benefit of an immunocytokine approach can be explained in part by mAb-induced target-specific cell death mediated via several indirect mechanisms: (1) immune effector cell-mediated antibody-dependent cellular cytotoxicity (ADCC); (2) complement-mediated cytotoxicity (CDC); (3) restoring immune effector cell function; and (4) direct mechanisms such as caspase-dependent apoptosis (Fig. 1). Indeed, the anti-CD38 mAbs inhibit immunosuppression exerted by regulatory T cells in MM [44–46] in addition to inducing myeloma cell death via lysosomal-associated and apoptotic pathways, which can be further enhanced by immunomodulatory drugs (IMiDs) [47]. Anti-CD38 mAbs may also inhibit MM-activated CD38+ pDC precursors [48] and/or restore DC maturation and presentation of tumor antigens, thereby further enhancing anti-tumor immunity. The addition of IFN-α was reported to augment ADCC by therapeutic mAbs both in vitro and in vivo [49, 50]. Specifically, mAb-mediated ADCC can be enhanced by IFN-α in the 3 ways: (1) enhancement of total target–mAb–effector binding by increasing tumor-associated antigen expression on tumor cells, as evidenced by in vitro studies showing that IFN-α induces CD20 upregulation on malignant B cells [51]; (2) activation of immune cells either directly, as IFN-α is a strong stimulus of NK cell activity, or indirectly through IFN-α-mediated upregulation of NKG2D ligands, which bind to co-stimulatory natural-killer group 2, member D (NKG2D) receptors expressed by NK cells, CD8 T cells, γδ T cells and macrophage [52]; and (3) blocking of effector cell ‘inhibitory’ signals, which remains largely unexplored. Additionally, type I IFN-containing immunocytokines can also target the tumor microenvironment by specifically binding to epidermal growth factor receptor on CTLs [53]. Taken together, cell-specific responses to tumor-targeting IFN-α-containing-mAbs relate to its sensitivity to IFN-α, the specific mAbs used, as well as the expression and density of the targeted tumor-associated antigens.

Prior to the discovery of anti-CD20 mAbs, anti-tumor cytotoxic effect of mAbs was limited. In 1984, the idea of using mAbs to deliver IFNs into specific cellular compartments was first proposed in a human cancer model to exploit the anti-Epstein-Barr viral and anti-proliferative effects of IFNs [54]. In 1993, the anti-tumor activity of an immunoconjugate comprising natural IFN-α bound to a mAb specific for a human breast epithelial membrane mucin was studied in a xenograft tumor mouse model [20]. This study highlighted the potential feasibility of antibody-based IFN-α fusion proteins. Since then, the introduction of newer and highly potent mAbs (such as rituximab/anti-CD20, daratumumab/anti-CD38, elotuzumab/anti-SLAMF7) has renewed interest in the development of IFN-α-targeted immunocytokines. Pre-clinical studies now evaluating anti-CD20-IFN-α and anti-CD20-IFN-β against B cell lymphoma, as well as anti-CD138-IFN-α against myeloma [17, 55–57]. Genetically engineered anti-CD20-IFN-α fusion proteins exert direct cytotoxicity and overcome CD20 mAb resistance in mice bearing B-cell lymphoma xenografts [58]. In MM, anti-CD138-IFN-α fusion proteins in combination with bortezomib resulted in synergistic cytotoxicity in a MM mouse model [59]. These preclinical studies form the rationale for the subsequent clinical trials [60]. Phase I clinical evaluation of anti-CD20-IFN-α to treat B-cell lymphomas (ClinicalTrials.gov Identifier NCT02519270) has been initiated and is still ongoing (Table 3).

**Table 3:** Current trends in human monoclonal antibody fusion proteins targeting IFNs in B cell malignancies

Targeted interferon-based therapy	Malignances under investigation	Research group/company	Clinical stage	References
Anti-CD38-IFNα	Multiple myeloma	Teva pharmaceuticals	Preclinical	Pogue et al. [60]
Anti-CD138-IFNα	Multiple myeloma	Division of Hematology and Oncology, Department of Medicine, UCLA (USA)	Preclinical	Vasuthasawat et al. [59]
Anti-CD20-IFNα	B cell NHL	Immungene (USA)	Pre-phase 1	Xuan et al. [57]
Anti-CD20-IFNα	B cell NHL	Immunomedics Inc (USA)	Preclinical	http://www.immunomedics.com
Anti-HLA-DR-IFNα	B cell lymphoma/leukemia, multiple myeloma		Preclinical	
Anti-HER2/neu-IgG3-IFNα	B cell lymphoma	University of California (USA)	Preclinical	Huang et al. [17]

*IFN* interferon, *NHL* non-Hodgkin lymphoma, *HLA-DR* human leukocyte antigen DR, *UCLA* the University of California, Los Angeles

In future, the ability to define patients who respond optimally to IFN-α**-**based immunotherapies is a central goal in cancer immunotherapy. Patients with B-cell lymphomas and MM are often immunocompromised, due to both the disease and its treatment. As IFN-α acts through activation of the immune system, a compromised immune system may limit IFN-α’s efficacy. In our opinions, IFN-α-based immunotherapies may benefit the following subpopulations of patients: (1) patients who have responded to intensive chemotherapy and stem cell transplantation, whose response may be deepened and prolonged by IFN-α-based immunotherapy; (2) patients with very early stage and/or indolent disease, limited tumor burden, and an intact immune response; (3) patients with robust anti-viral immunity, which can be reprogrammed to target cancer instead; and (4) patients with highly detectable proportions of circulating immune effector cells. The search continues for other potential biomarkers of response to IFN-α-based therapies, while genome-wide gene expression profiling (GEP) has in recent years emerged as a powerful tool. Taken rheumatoid arthritis for example, GEP revealed that pharmacodynamic differences in anti-CD20 mAb response very closely correlate to type-I IFN response gene activity [61]. Specifically, the increased expression of a set of 6 IFN response genes (RSAD2, IFI44, IFI44L, HERC5, LY6E and Mx1) was associated with a good response to mAb-based immunotherapy while higher baseline levels of type I IFNs may predict for lack of response to anti-CD20 mAbs.

## Conclusions

The unique and multi-faceted anti-tumor mechanism of mAb-targeted IFN-α-based immunotherapy makes it a very promising agent for treatment of B cell malignancies. Moving forward, in vitro and in vivo preclinical studies are needed to further evaluate the therapeutic efficacy of mAb-targeted IFN-α-based immunotherapies both as monotherapy and in combination with other MM therapies including proteasome inhibitors, immunomodulatory drugs, and glucocorticoids. The following questions can be examined in preclinical models: (1) the relative anti-MM activity of intrinsic INF-α and anti-IFN-α mAb; (2) the reduction of tumor burden, including malignant stem cells, triggered by anti-IFN-α mAb compared to mAb alone; (3) impact of IFN-α on expression of tumor associated antigens (either on tumor cells or cells the within immune regulatory network) targeted by mAbs: by increasing the expression of CD20 on malignant B cells [51], anti-CD20-IFN-α has shown promising anti-tumor activity in patients who were unresponsive to anti-CD20 mAb-containing regimens; (4) tolerability of mAb-targeted IFN-α-based immunotherapies compared to systemic administration of IFN-α; and (5) whether mAb therapies modulate the IFN pathway. Additional studies are required to determine the optimal doses, schedules, and sequence of mAb-targeted IFN-α-based immunotherapies. Current research provides a strong rational for the early clinical evaluation of these agents. Ultimately, the clinical utility of these targeted IFN-based approaches will need to be validated in multicenter, randomized-controlled prospective studies.

## Additional file

**Additional file 1: Table S1.** History of IFN-α treatment in human B cell malignancies. Abbreviations: IFN, interferon; ND, newly diagnosed; RR, relapsed and/or refractory; MCL, mantel cell lymphoma; SLL, *small lymphocytic lymphoma; FL, follicular lymphoma; DLBCL, diffuse large B cell lymphoma; NHL, non*-*Hodgkin lymphoma; MALT,* mucosa-associated lymphoid tissue; HL, Hodgkin lymphoma; pcMZL, primary cutaneous marginal zone *lymphoma; CLL, chronic lymphocytic lymphoma; AITL, a*ngioimmunoblastic T-cell *lymphoma;* PTCL, peripheral T cell lymphoma; DLCL, diffuse large cell lymphoma; MM, multiple myeloma; WM, Waldenstrom’s macroglobulinemia; NK, natural killer; LG, *large granular; IL, interleukin;* HDT, high dose treatment; ASCT, *autologous stem cell transplant;* ABSCT, autologous bone marrow or blood stem cell transplantation; PBSCT, peripheral blood stem cell *transplantation;* CHOP, cyclophosphamide, vincristine, doxorubicin, and *prednisone; UA, unavailable;* *IFN Dose: high-dose IFN (5 MIU/m^2^ daily), low-dose (3 MIU/m^2^/day, 3 times a week), intermediate-dose, between high and low doses.

## Abbreviations

mAb: monoclonal antibody
FDA: Food and Drug Administration
MM: multiple myeloma (MM)
SLAMF7: SLAM Family Member
PFS: progressive-free survival
CSCs: cancer stem cells
MRD: minimal residual disease
IFN: interferons
IFN-α: Interferon-alpha
CTCL: cutaneous T cell lymphoma
DCs: dendritic cells
IPCs: IFN-producing cells
IFN-AR: IFN-α receptor
STAT: signal transducers and activators of transcription
CTL: cytotoxic CD8+ T cell
Th1: T helper-1
PD-1: programmed cell death-1
NHL: non-Hodgkin’s lymphoma
FL: follicular lymphoma
ADCC: antibody-dependent cellular cytotoxicity
CDC: complement-mediated cytotoxicity
IMiDs: immunomodulatory drugs
NKG2D: natural-killer group 2, member D
GEP: gene expression profiling
